# Detecting Silent Vocalizations in a Locked-In Subject

**DOI:** 10.1155/2013/594624

**Published:** 2013-11-07

**Authors:** Elina Sarmah, Philip Kennedy

**Affiliations:** ^1^Georgia Institute of Technology, Atlanta, GA 30313, USA; ^2^Neural Signals Inc., Duluth, GA 30096, USA

## Abstract

*Problem Addressed*. Decoding of silent vocalization would be enhanced by detecting vocalization onset. This is necessary in order to improve decoding of neural firings and thus synthesize near conversational speech in locked-in subjects implanted with brain computer interfacing devices. *Methodology*. Cortical recordings were obtained during attempts at inner speech in a mute and paralyzed subject (ER) implanted with a recording electrode to detect and analyze lower beta band peaks meeting the criterion of a minimum 0.2% increase in the power spectrum density (PSD). To provide supporting data, three speaking subjects were used in a similar testing paradigm using EEG signals recorded over the speech area. *Results*. Conspicuous lower beta band peaks were identified around the time of assumed speech onset. The correlations between single unit firings, recorded at the same time as the continuous neural signals, were found to increase after the lower beta band peaks as compared to before the peaks. Studies in the nonparalyzed control individuals suggested that the lower beta band peaks were related to the movement of the articulators of speech (tongue, jaw, and lips), not to higher order speech processes. *Significance and Potential Impact*. The results indicate that the onset of silent and overt speech is associated with a sharp peak in lower beta band activity—an important step in the development of a speech prosthesis. This raises the possibility of using these peaks in online applications to assist decoding paradigms being developed to decode speech from neural signal recordings in mute humans.

## 1. Introduction

Locked-in syndrome (LIS) is a clinical condition in which subjects suffer from complete paralysis and cannot speak but are awake and cognitively intact. This syndrome results from pontine ischemic or hemorrhagic strokes, amyotrophic lateral sclerosis (ALS), and other etiologies. It has been a long-term goal for many researchers to provide these subjects with a means of communication. Currently, assistive communication for locked-in individuals can be achieved via various devices such as external or EMG switches [1], EEG [2], ECOG [3], or by using implanted electrodes within the brain [4–7]. The external noninvasive methods to produce speech output are inherently slow, with speech sounds being produced from a computer speaker after the subject has slowly spelled out what he/she wants to say. Decoding of neuronal activity from the cortical speech area is more likely to provide a more natural communication rate, perhaps approaching conversational speed. Efforts to decode speech phonemes from a locked-in subject using single unit activity have been partially successful to date [4]. A specific roadblock remains the real-time detection of the onset of attempted vocalization. If vocalization onset could be detected, then decoding of the neural signals would be simplified because it would allow pattern matching of relevant data segments, ignoring irrelevant data segments, and in that way it would minimize the choices inherent in neural net paradigms.

Our purpose in using the data recorded from the locked-in subject was to determine if potential changes in the frequency band from intracortical recordings could serve as markers for the onset of vocalization. In general, movement-related cortical potentials have been studied for many years [8], and sources of movement-related cortical potentials have been somatotopically defined [9]. Most of the available studies have utilized averaging techniques to analyze these potentials, but it has now become possible to detect movement-related cortical potentials on a single-trial basis through the use of optimized spatial filtering techniques [10]. We chose to focus on the lower beta band (14–20 Hz) since this band has been associated with event-related synchronizations (ERS) [11]. Most studies, however, have demonstrated postmovement ERS, not premovement. However, a study by Grin-Yatsenko et al. [12] demonstrated increases in spectral power within the 14–20 Hz band in long-term depressed subjects. They concluded that enhancement of beta power may correlate with anxiety symptoms that most likely play an important role on the onset of depressive disorder. Perhaps the beta peak reported here corresponds to a mild anxiety situation inherent in speech onset in response to a computer command. An alternative view, and a more convincing one, relates to Basal Ganglia oscillations where Leventhal and colleagues [13] demonstrated brief beta oscillations around 20 Hz that “reflect a post decision stabilized state of cortical-BG networks, which normally reduces interference from alternative potential actions. The abnormally strong beta seen in Parkinson's disease may reflect over-stabilization of these networks, producing pathological persistence of the current motor state.” We suspect that the findings below reflect a stabilized state prior to the motor task of vocalization.

The data presented here was recorded from a mute subject who was chronically implanted with a Neurotrophic Electrode within his primary or premotor speech or articulatory cortex (face area) [4]. We find that increases in the lower beta band power in continuously recorded intracortical signals are likely to occur close to vocalization onset. Subsequent analyses sought to determine how close to the onset of attempted vocalization the beta band increases occurred since proximity to speech onset is obviously key. In addition, the minimal data segment length in which lower beta band peaks could be detected is important to minimize delay in speech production.

The implanted mute subject is unique with no other subjects being now available. Thus to attempt confirmation of the results from this individual, his lower beta band analyses were compared with lower beta bands detected from recorded EEG signals in three intact speaking humans. Clearly, no exact comparison could be made since the speaking subjects did not have cortical implants. Such confirmation was needed since a data from a single subject requires some kind of validation, and it is impossible to know if the mute subject was in fact trying to vocalize or if the lower beta band peaks might have been random events. Finally, we sought to determine if the lower beta band peaks, if found, were related to higher order speech processing or to output phases of the speech as implied by movements of the articulators. Our overall aim was to develop a method of identifying lower beta band peaks in real time with a view to optimizing the decoders for detection of attempted speech in mute, locked-in subjects.

## 2. Methods

### 2.1. Recordings in the Mute Patient

Recordings of neural activity were performed in a locked-in patient, ER, who sustained a traumatic basilar artery thrombosis and resulting brainstem stroke at age 16 years, 10 years prior to these studies. He was taking no medications during these recordings or occasionally Provigil (Modafinil), a wakefulness medication administered 20 minutes prior to the recordings. The recordings were made possible with the surgically implanted Neurotrophic Electrode. The complete description of the components and assembly of the Neurotrophic Electrode has been detailed in a previous publication [14]. The recordings are bipolar from within the cone tip of the electrode, using Teflon insulated gold wires that are 400–500 *μ*m apart. This ensures that the recorded signal is free from artifacts such as line noise or spurious EKG or EMG components. Electrical potentials can be recorded several weeks after the implantation of the electrode and stabilize within 3 to 4 months. As previously detailed [15, 16], the implanted custom-made amplifiers, implanted on the skull adjacent the electrode, have gains of 100x and high and low pass filters (4–4,000 Hz). The resulting electrical signals modulated a 30–50 MHz FM signal for transcutaneous transmission. After further 1000x amplification, the signals were archived on digital tape at a sampling rate of 20 kHz. 

**Figure 1:**
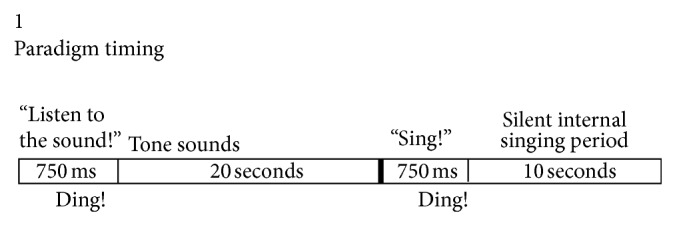
The paradigm shows that prior to silent speech or silent singing, there is a 10 or 20 second period of listening to the sound of the phoneme or tone.

Figure 1 shows the timing of typical recording sessions. Figure 1 shows that once recording began, the locked-in subject was instructed by a computer to listen to a phoneme for a 4-second period or a tone for a 20-second period. The subject was then given a computer-generated signal to “Sing,” (followed by a “ding” sound). The “ding” sound started the “speak” or “sing” period, which gave the subject 7 seconds to internally produce the phoneme or 10 seconds to internally sing (or hum) the tone that was just heard.

**Figure 2:**
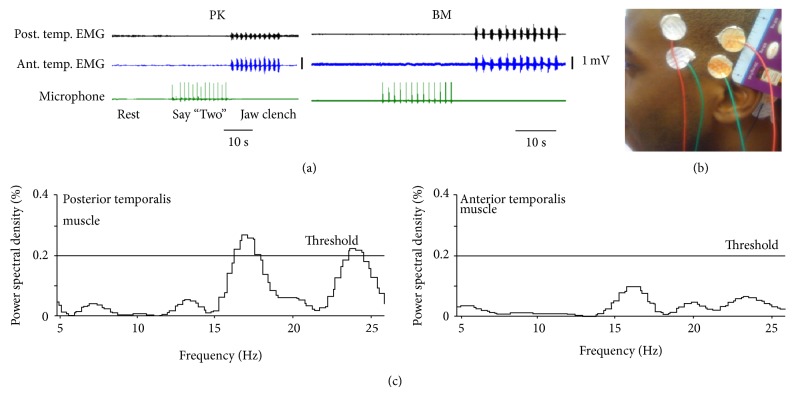
(a) Paradigm for sampling from anterior and posterior temporalis muscle EMG with three conditions, rest, say “two”, and jaw clench. (b) Photos of electrode in position over the speech area underlying the posterior part and anterior part of the temporalis muscle, with the reference electrode over the mastoid bone behind the ear. (c) An example of a lower beta band peak detected in the EEG/EMG signal is shown. Note the threshold of 0.2% PSD that is crossed only by the signal over the posterior temporalis muscle that overlies the speech cortex.

For every trial of attempted vocalization, the 7–10 seconds of data following the speak marker were evaluated. This was performed by examining each second of data with respect to its power spectral density percentages (PSD%) of the frequencies between 5 and 25 Hz using NeuroExplorer Software (NEX, from Plexon Inc., Dallas, TX). The PSD% was calculated as a percentage of the amplitude of the spectral analysis. In order to determine the limit at which power spectral changes could be detected, the calculations were repeated with shorter Fast Fourier Transform data segments (utilizing 500 ms, 250 ms, 150 ms, 100 ms, and 50 ms of data). The FFT program provided visual examination of the data to determine segment selections. The PSD% and center frequencies of the lower beta band peaks were then recorded at the minimum time segment at which the peak was still visible (rarely as short as 50 ms). This process was repeated for each second of the entire 7- or 10-second interval. Once the Speak or Sing period for every trial was examined, the 20-second listen periods (prior to the “ding”) were then evaluated using the same process. Data were collected for latency of data segment from “Ding”, average PSD%, the average frequency between 14 and 20 Hz at which the peak was detected and average minimum data segment needed to detect the peak. At this initial stage of data collection, the lower beta band peak was accepted if it was at least twice the amplitude of the surrounding baseline. To tighten this criterion, subsequent analyses required a 0.2% increase in PSD% as illustrated in Figure 2 for the EEG data collection. Cross-correlations of single unit firing rates were performed using the NEX software using a specified unit as the index unit and examining the firing of other units over a 5-second data segment in 100 ms bins to determine if they were correlated to the index unit.

### 2.2. Recording Nonmute Subjects

Standard EEG and EMG recordings were performed during vocalization in three nonmute subjects, ES, BM, and PK (ages 20, 34, and 63 years, resp.) to determine if lower beta band peaks could be detected from the EEG signal during speech. The paradigm is shown in Figure 2(a) with 20-second rest periods followed by the say period consisting of 10 to 12 attempts to say “two” and then 10 to 12 attempts at jaw clenching to activate EMG. The “two” sound was chosen because it is mainly a tongue movement and would thus minimize temporalis muscle EMG contamination of the EEG signals. The extent of such contamination was determined by concomitant recordings of anterior temporal EMG signals. A rest period completed the controls.

All subjects were left hemisphere dominant. Two EEG electrodes were therefore secured to the scalp on the left side of the head over the speech motor cortex, using water-soluble EEG paste (EC2 Electrode Cream from Grass Technologies, distributed by Astro-Med Inc., West Warwick, RI) as shown in Figure 2(b). One electrode was placed at the point where the upper superior/anterior edge of the ear meets the scalp and the other 1 inch above the first in line with the vertex (Figure 2(b)). In subjects PK ((a), left panel) and BM ((a), right panel), a second set of electrodes, acting as controls, were placed at the anterior edge of the temporalis muscle at the hairline as shown in the figure. For all recordings, the reference electrode was placed over the left mastoid bone. Amplifier gains (BMA-200 or BMA-831 from CWE Inc., Ardmore, PA) were set at 200x, and the signals bandpass-filtered with filter settings of 1–10,000 Hz. The subjects spoke the word “Two” for a period of 10 to 12 trials, with 1.5 to 2 seconds in between each trial. Control recordings taken during the same sequence included [1] EMG activity from the anterior part of the temporalis muscle that was not over the assumed speech cortex, [2] intermittent rest periods of 20 seconds where the subject sat motionless and was not speaking, and [3] jaw clenches that specifically activated the temporalis muscle and, thus, resulted in EMG activity, with the expectation that lower beta band peaks would not be detected over the anterior temporalis but would be detected over the posterior temporalis muscle that overlies the portion of the speech cortex involved in the control of articulators. To detect lower beta band peaks, a 200 ms window was moved across 1 second of data in 100 ms steps. The 1 second of data was chosen around the time of microphone detection of vocalization about 500 ms before and 500 ms after. If a lower beta band peak was suspected, wider and narrower data segments were also examined. The 0.2% increase in PSD was chosen empirically during data perusal.

## 3. Results

Data from the mute subject and the normal controls were analyzed in three phases. Phase 1, mute subjects: increases in amplitude of lower beta band peaks were determined during attempted silent production of individual phonemes (1a) and silent singing (1b). Phase 2: cross-correlation analyses were explored to determine functional implications of the results in phase 1. Phase 3, nonmute subjects: since vocalization attempts could not be independently confirmed in another mute subject, speaking subjects were tested for the possible presence of beta peaks associated with speaking.

### 3.1. (Phase 1a) Analysis of (Presumed) Phoneme Detection in the Mute Subject

**Figure 3:**
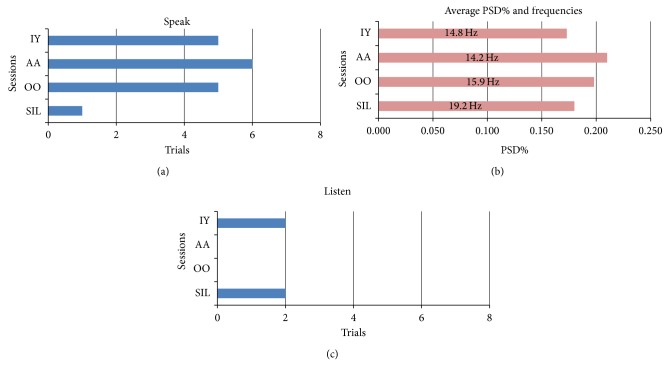
Upper panel illustrates the number of lower beta band peaks found in the trials for different vowel phonemes and during silent trials during internal vocalization of phonemes. The middle panel illustrates the average PSD% and frequencies at which the beta peaks occurred. The third panel illustrates the number of trials with lower beta band peak frequencies during the “listen” periods. Data from 2007-10-09 file no. 07.

Lower beta band peaks were evident in the majority of phoneme production trials as shown in Figure 3. There were a total of 7-8 trials for each of the phonemes, IY, AA, OO, and Silence which served as control. These phonemes were chosen primarily because their fundamental frequencies are well differentiated (high frequency for “IY,” e.g.). If the phoneme frequencies were close together, then lower beta band peaks for one particular phoneme could not be claimed to be specific for that phoneme. Lower beta band peaks were present in 5 of 8 trials for the IY and OO phonemes, 6 out of 7 AA trials, and only 1 of the 8 Silence trials. Using Fisher's exact two-tailed test, statistically significant differences were found (Fisher exact test, normalized, two-tailed, *P* = 0.0001) when comparing between each normalized phoneme value and the silent control. To account for the small sample size, a nonparametric test was also conducted. Statistical significance was also observed using the Mann-Whitney test (*P* = 0.025) to compare the ratio of successful to unsuccessful trials between phoneme and silent control. 

To compare the strength of lower beta band peaks, power spectral densities were analyzed using the amplitude of the signal expressed as percentages of total power (PSD%). The middle panel of Figure 3 shows little difference in amplitudes, that is, strength. During the IY trials, the average PSD% was 0.173% at an average peak frequency of 14.8 Hz at the minimum time segment. For the OO phoneme, the average PSD% was 0.198% at a peak frequency of 15.9 Hz. The strongest lower beta band peaks occurred in the AA phoneme, with an average PSD% of 0.21 and peak frequency 14.2 Hz. For the one peak found in the Silent segment, the PSD% was 0.180 at 19.2 Hz (a frequency that was notably different from the average phoneme frequency of 14.9 Hz). The latency of the lower beta band peaks after the speak marker ranged between 1.75 and 4.63 seconds. IY at 4.63 seconds after the marker, OO at 3.85 seconds after the marker, AA at 2.75 seconds after the marker, and the one Silent control peak occurred at 1.75 seconds after the speak marker. 

As an additional control, the *listen* period that preceded each speak period was examined. Out of the total 8 trials for each phoneme, only 2 trials from the IY phoneme and 2 trials from silence had evidence of lower beta band peaks (lower panel Figure 3). These were significantly different from the Speak period data (Fisher Exact test, normalized, two-tailed, *P* = 0.0001). These peaks occurred within the first second after the marker, and the average PSD% and peak frequencies for the IY phoneme and silence were 0.182 at 14.8 Hz and 0.111 at 18 Hz, respectively. 

These results, including number of peaks, average PSD%, average frequency, and average minimum time segment are summarized in Table 1.

### 3.2. (1b) Analysis of (Presumed) Onsets of Tone Reproduction (Singing)

Having found that lower beta band peaks in silent “Speak” periods were significantly more frequent than in Silent controls or Listen periods, we hypothesized that lower beta band peaks may also occur during other types of vocalizations such as silent “singing.” Archived data were therefore examined in which the subject listened to a tone and then hummed or sang the tone in his head beginning as soon as he heard the “ding” signal. Single-unit responses from these experiments [16] demonstrated that coordination of single unit firings in response to silent singing was improved with the introduction of feedback (as elucidated in Section 4). 

**Figure 4:**
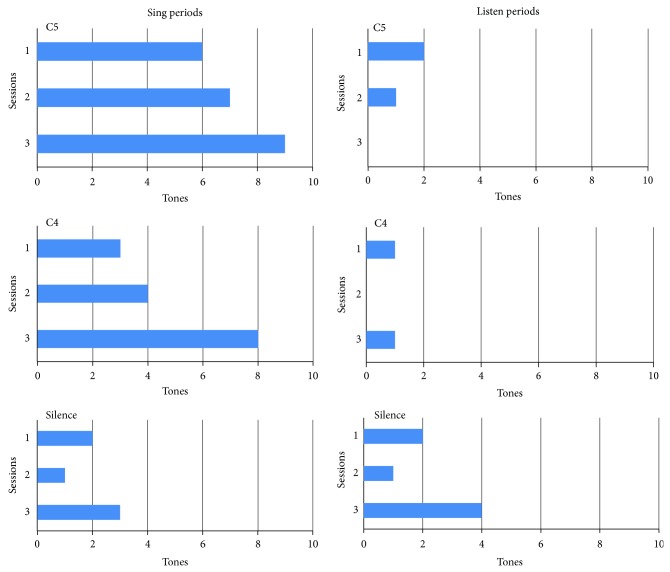
Number of trials with lower beta band peak frequencies during internal singing at two different tones C4 (256 hz) and C5 (512 hz). The controls include the silence (bottom left) randomly presented during singing data collection and the listen periods (right side panels). Data is taken from dates 2009-3-30 (session 1: 1949 days after implantation), 2009-4-03 (session 2: 1953 days after implantation), and 2009-04-06 (session 3: 1946 days after implantation).

Changes in the firing frequency of single units in response to the tones C5 (high C, 528 Hz) and C4 (middle C, 256 Hz) were analyzed along with silent periods as controls on days 1549, 1553, and 1556 after implantation. These data revealed a gradual increase over the three sessions of the number of lower beta band peaks present in each session of the C5 and C4 tones (Figure 4). Out of a total of 10 trials for each tone, 6 of the C5 trials contained lower beta band peaks on Session 1, 7 on Session 2, and 9 on Session 3. For the C4 tone, 3 of the trials contained lower beta band peaks on Session 1, 4 on Session 2, and 8 on Session 3. The Silence trials showed only 1–3 lower beta band peaks each session. Similar to the phoneme data, the Listen periods on these days had evidence of very few lower beta band peaks. Each trial contained only 1–4 peaks (Figure 4). Session 2 of C4 and Session 3 of C5 showed no lower beta band peaks at all during the listen period. The PSD% of these lower beta band peaks was between 0.309–0.818 at a mean frequency of 16 Hz.

The PSD% of the lower beta band peaks found in the Sing data was much stronger than those of the phoneme data. The combined PSD% averages of the phoneme data were only 0.190%, whereas the combined average for the tones data was 0.559%. The PSD% for both the C5 and C4 tests fell in the range of 0.400–0.700 for all 3 days at a mean frequency 15.2 Hz. The latency of the lower beta band peaks present was between 1 and 5.5 seconds after the sing marker. These results, along with the average PSD%, average frequency, and average minimum time segment at which the lower beta band peak was visible, are shown in Table 2.

### 3.3. (Phase 2) Functional Correlations of Beta Peaks

**Figure 5:**
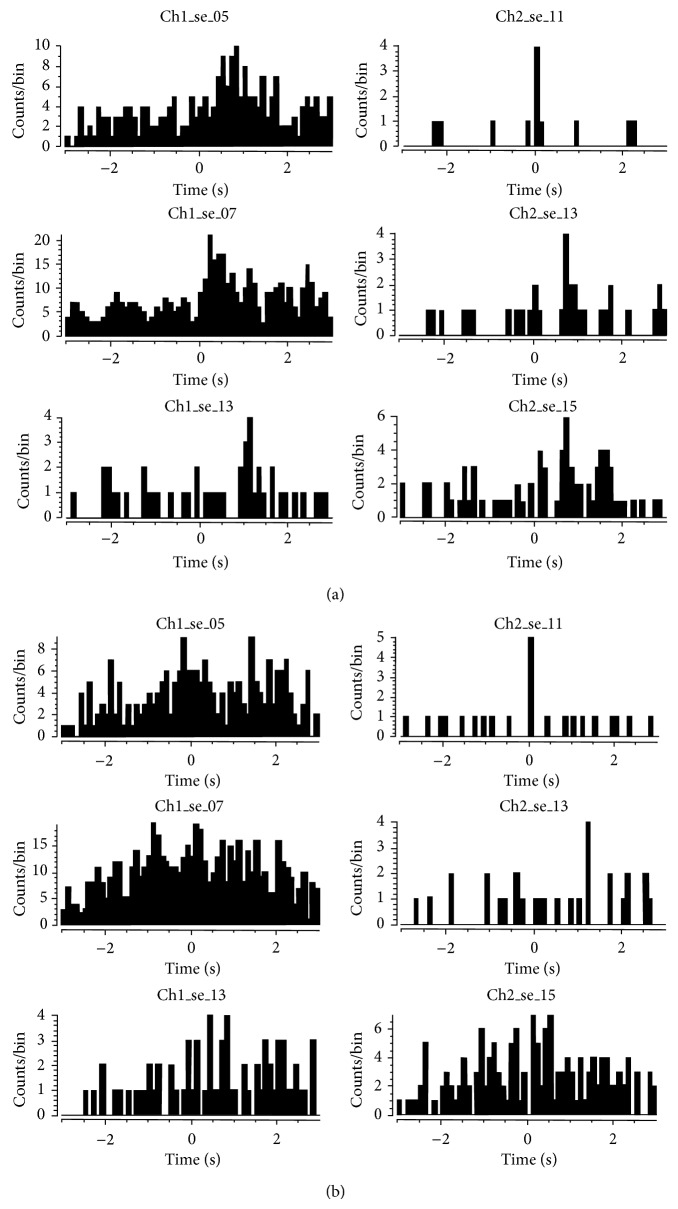
Cross-correlations: data is from session 3 (day 1556, May 5, 2009) when loudness feedback (the speaker output volume increased in direct proportion to the firing frequency) was provided and synchronized single unit firing was optimal. Reference unit is Ch2_se_11 (top right panel). (a) The data segment shown in this figure was recorded 5 seconds *after* a local field potential spectral peak in the beta band. (b) This data segment was taken from a period of 5 seconds *before *the local field potential spectral peak in the lower beta band. (Lower beta band peak is @ 4346.55 seconds into file from day 1556.) Note the improved cross-correlations *after* the beta peak in panel (a). Data are selected from two channels, with 31 units in total.

If lower beta band peaks predict the onset of speaking or singing as shown in the preceding data, then there ought to be a functional change in neuronal firing rates after the onset of the peak compared with before the peak. In other words, lower beta band peaks should predict greater coherence of firing rates. Of relevance is that prior studies [16, 17] indicated task-related increases in cross-correlations. We reexamined the single-unit spiking data to determine if there were differences in coherence before and after the detection of lower beta band peaks. We found that the cross-correlation values were increased in the 5-second data segment following the lower beta band peak in the Sing period, compared to the cross-correlation values in the prelower beta band peak period for some units (Figures 5(a) and 5(b)).

**Figure 6:**
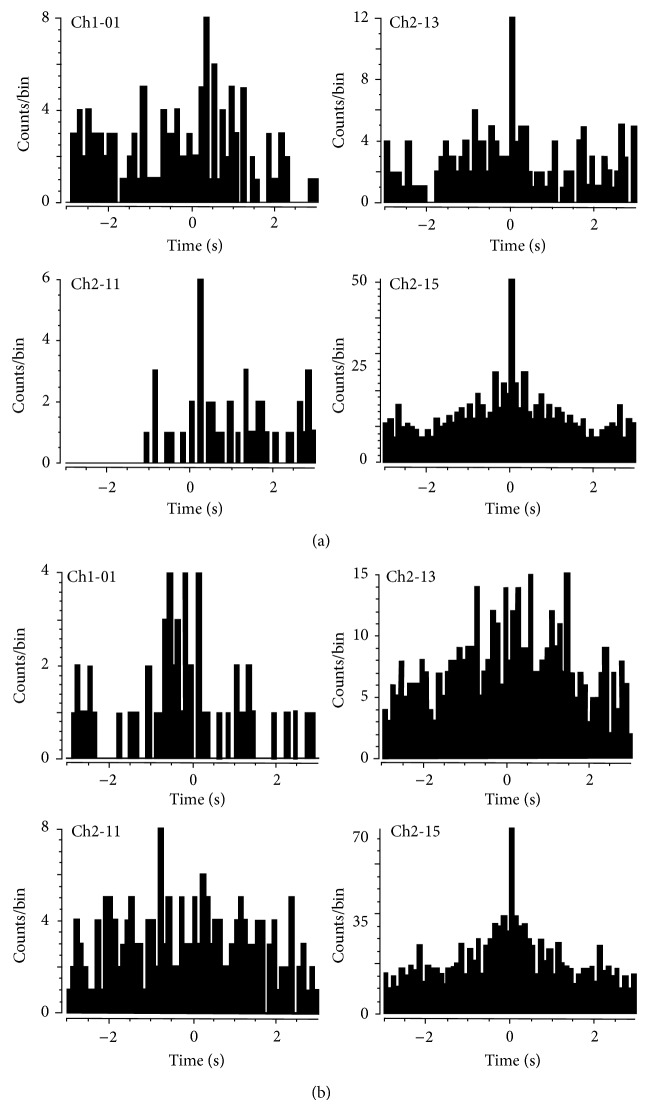
Cross-correlations: data is from session 3 (day 1556, May 5, 2009) when loudness feedback was provided and synchronized single unit firing was optimal. Reference unit is Ch2_se_15 (bottom right panel). (a) Data segment is 5 seconds *after* lower beta band peak. (b) Data segment is 5 seconds *before *lower beta band peak. Both data segments are from a Sing period (@ 4338 seconds). Note the improved cross-correlation *after* the lower beta band peak in panel (a).

A further control on these data was considered necessary because the lack of cross-correlations within the data before the LFP lower beta band peak may have been due to the Listen function per se. Recall, however, that very few lower beta band peaks were detected within the Listen periods. An occasional lower beta band peak occurred near the middle of the sing period (presumably because he delayed silent singing). Thirty-one single units were separated from two channels of multiunit data. These data before and after these lower beta band peaks were therefore used as further controls. The data in Figure 6 uses single unit Ch2_se_15 as the reference. The result is essentially the same as previously found with cross-correlation being more evident *after* the beta band peak, compared to before the beta band peak.

### 3.4. (Phase 3) Detection of Lower Beta Band Peaks in EEG Signals during Vocalization in Nonmute Subjects

To examine whether lower beta-band activity might also occur in nonmute, speaking subjects, data were collected from three subjects (ES, BM, and PK), using the paradigm described in methods (Figure 2(a)). Epochs of 10 to 12 trials consisted of six for PK, three for BM, and one for ES. The two pairs of external electrodes were placed over the same muscle, namely, the temporalis, with the posterior electrodes being positioned carefully over the speech area and the anterior electrode pair clearly anterior to this area as shown in Figure 2(b). Standard neurosurgical landmarks were used to locate the assumed speech motor area which controls the speech articulators. An example of a lower beta band peak seen in the EEG/EMG signal is shown in Figure 2(c). Lower beta band peaks with an amplitude exceeding 0.2% were empirically considered as possibly significant.

**Figure 7:**
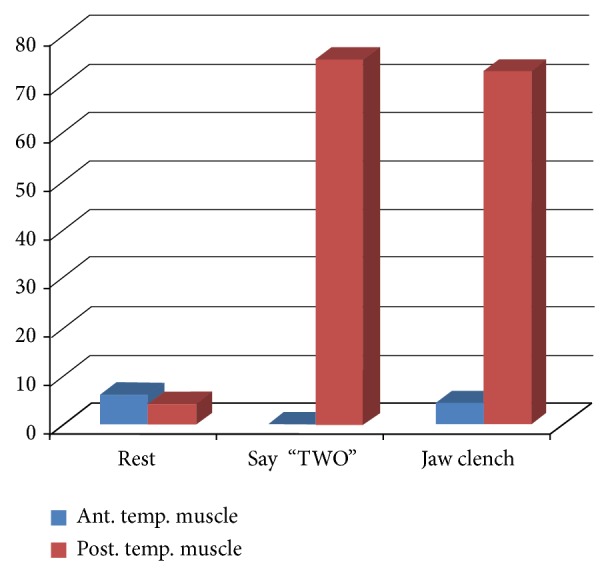
Lower beta band peaks associated with three different testing conditions. During the rest period, virtually no beta peaks were detected. During the speaking of “Two” beta peaks appeared over the posterior part of the temporalis muscle (that overlies the speech area), but not the anterior (that is not over the speech area). During articulatory movement of jaw clenching, beta peaks were observed similar to the say “Two” data collection, namely, over the posterior part of the temporalis muscle, not the anterior, indicating that articulator movements are related to beta peaks.

Lower beta band peaks were detected in all subjects from the posterior but not the anterior muscle (Figure 7) for columns “say Two,” and these results were statistically significant (Fisher test, two-tailed, *P* = 0.0001, pooled data with similar significance levels for each subject). The illustrated data were obtained from subjects PK and BM. Subject ES had 100% detection of lower beta band peaks in 20 trials but did not have the control data, so those results are not illustrated. The results in the “Say Two” pair of columns in Figure 7 suggested that lower beta band peaks could have come from the motor speech area but possibly might have been present whether speaking or resting. Thus 20 seconds of rest were analyzed as shown in the middle block of data in Figure 7 by moving the detection window of 200 ms data segments in 100 ms steps over the full 20 seconds. Very few lower beta band peaks were detected from electrodes overlying the anterior or posterior parts of the muscle during this control rest period. The few lower beta band peaks detected from the anterior and posterior parts of the temporalis muscle were not significantly different from each other (Fisher test, two-tailed, *P* = 0.0001).

The lower beta band peaks detected over the speech area that was assumed to underlie the posterior temporalis electrode could have arisen either as a result of higher order speech processing (syntax, word, or sentence processing) or could have been due to movement of the articulators. To distinguish these options, the subjects produce jaw clenches which activated both anterior and posterior parts of the temporalis muscle without making any attempt to speak or imagine speech. This movement requires movement of the articulators but clearly not a movement involved in speech production. Lower beta band peaks were detected over the posterior temporalis muscle, not the anterior, as shown in Figure 7 for “jaw clench.” The lower beta band peaks detected during jaw clenches and the frequency during say “two” (tall blue columns) were not significant different (Fisher test, 2 tailed, *P* = 0.1201). Thus, lower beta band peaks were evident during activation of the articulators when the subject was speaking and when clenching the jaw. This result suggests a strong association between lower beta band peaks and the use of articulators but not between lower beta band peaks and higher order speech processing.

### 3.5. Latency between Lower Beta Band Peaks and Vocalization Onsets

**Figure 8:**
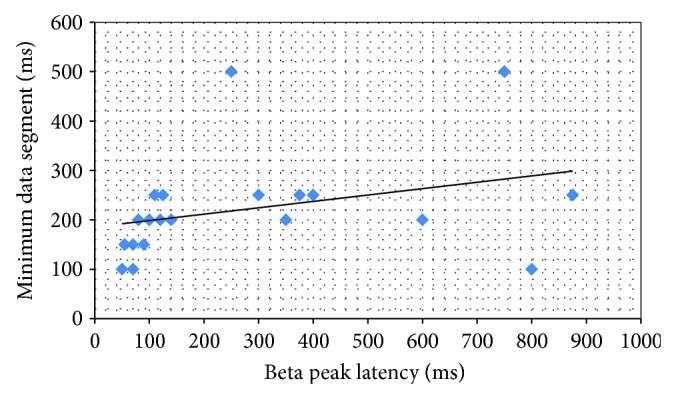
Lower beta band peak latency and minimum data segment length (in ms) at which beta peak could be detected. Note the cluster of values between 50 and 150 ms with respect to the peak latency and the minimum data segment required to detect the phoneme or sing between 100 and 250 ms.

Lower beta band peaks at the beginning of vocalization might be used as trigger signals for computer-generation of speech output in clinical applications (as described in Section 1). For this reason, we were interested in determining the shortest latency between the detection of the lower beta band peaks and vocalization onset as determined by the microphone output. These values were plotted along with the shortest data segment length required to detect a lower beta band peak and shown in Figure 8. The average lower beta peak latency was 285 ms and the average minimum data segment length was 222 ms. Note, however, the strong cluster of latencies between 50 and 150 ms latencies and the data segment clusters between 100 and 250 ms. The average PSD% of these peaks was 0.201 at an average frequency of 19 Hz.

## 4. Discussion

The above data provide evidence that transient increases in amplitude of the power spectral density between 14 and 20 Hz (in other words lower beta band peaks) may be useful indicators of the onset of actual or silent movement of the articulators involved in both audible and silent speech. The evidence is obtained from recordings from the speech motor cortex of a locked-in subject making silent attempts to produce speech and from recordings made over the presumed speech area in speaking subjects.

The mute subject attempted to produce single phonemes and to sing (or hum) specific tones. The phoneme data in Figure 3 illustrate that lower beta band peaks occurred in intended productions of phonemes IY, OO, and AA but not in the Listen or Silent Control periods. A similar finding appeared in the Sing data in Figure 4 where lower beta band peaks were detected during intended production of C4 and C5 tones but not in the Listen or Silent control trials. This evidence is similar to the data showing that lower beta band peaks are detected prior to limb and mouth movements [8–10]. Note that the presently described lower beta band peaks are not identical to the frequency range described by others who chose anywhere from 14 to 30 Hz [17]. If lower beta band peaks are functionally relevant, there ought to be changes in firing activity of the underlying neurons. Evidence for functional relevance is shown in Figures 5 and 6. Functional relevance has already been demonstrated in single unit data recorded during phoneme production using cross-correlation techniques [18]. In the present data, cross-correlation values were lower before the lower beta band peak compared with after the peak (Figure 5). Since the data before the lower beta band peak were acquired during the Listen period and the Listen period was known to have fewer cross-correlations or Xcorrs, we also used a period during silent speech production where the lower beta band peak occurred a few seconds into the period (presumably, the subject delayed his speech attempt). There were fewer Xcorrs before the lower beta band peak compared to after the peak (Figure 6). These data provide evidence for functional relevance of the lower beta band peak.

There is no other speech cortex implanted human and unlikely to be any in the near future. Therefore, to verify these findings, lower beta band peaks were studied in speaking humans. External electrodes were placed over the area implanted in the mute subject, namely, the area of primary motor cortex that controls the articulators, otherwise known as speech cortex. This involved recording some EMG as well as, expectantly, EEG signals. It is worth noting that EMG contamination could not have occurred in the implanted mute subject because the recordings were made between bipolar wires within the 1 mm glass cone tip implanted within cortex, and with glass being an electrical insulator, only activity inside the glass tip can be recorded between the wires. With external electrodes, of course, a control was needed. The temporalis muscle is activated when speaking, chewing, and clenching the jaw. The anterior fibers are not over the speech area whereas the posterior fibers are. Thus, the anterior fibers can act as controls on the underlying speech area since both anterior and posterior are involved in the same function. As shown in Figure 7, the normalized data illustrate lower beta band peaks in the posterior part of the temporalis muscle during speech far in excess of the few found in the anterior part. Because these lower beta band peaks could have been randomly generated, a further control was needed to ensure that these were not “background noise.” As Figure 7 illustrates, there were very few lower beta band peaks during rest from either the anterior or posterior parts of the muscle suggesting that the lower beta band peaks seen during speaking were due to the subjects producing the phoneme. The question then arose as to whether or not the lower beta band peaks were produced by higher order speech functions or simply by movement of the articulators. Articulatory movements without speech were produced by clenching the jaws. Lower beta band peaks were observed over the posterior part of the muscle, but not the anterior part, as the third block in Figure 7 illustrates. These data provide evidence that the lower beta band peaks are associated with articulatory movements, whether or not the articulators are being used for speech production, implying that they are not associated with higher order speech processing.

**Figure 9:**
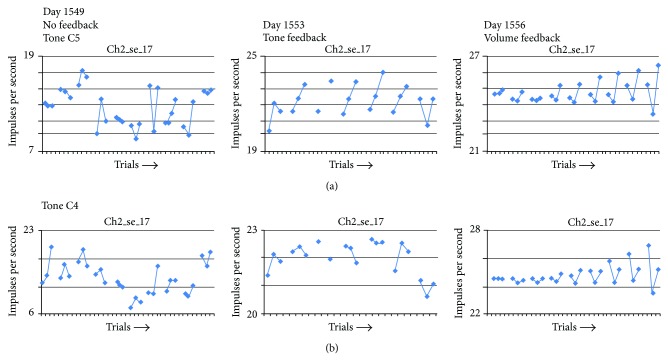
Data from unit ch2_17 followed over three sessions demonstrating the firing rate tuning over that time period with no feedback (day 1549), unit feedback (day 1553), and feedback of volume directly related to unit firing frequency (day 1556). Data for tone C5 @ 523 hz is illustrated in the upper panel. Similar data from days 1549, 1553, and 1556 for tone C4 @ 262 hz are illustrated in the lower panel. These changes paralleled the appearance of the lower beta band peaks illustrated in Figure 4.

These results raise several questions. One question is the possibility that the appearance of lower beta band peaks may be related to performance and specifically that an increase in frequency of lower beta band peaks might parallel an improvement in performance. An example is the gradual increase in lower beta band peak frequency in the Sing data over the three sessions illustrated in Figure 4. A precedent for this observation was noted in the analysis of the changes in the patterns of firing rates of single units in these same three sessions [16]. Unit ch2-17 illustrates this point in Figure 9. The values are grouped in three: the first is the average rate during Listen, the middle is the rate during a Control recording (when the subject sat quietly), and the third is the rate during silent Sing. During session 1 (day 1549 after implantation) firing rates were neither smooth nor symmetrical from trial 1 to trial 10. During session 2 (day 1553) firing rates began to appear more uniform and smoother. During session 3 (day 1556) firing rates were smoother and more symmetrical as the trials progressed. These intersession changes in firing rate symmetry appear to correlate with auditory feedback. No feedback was provided during session 1. During session 2, audible feedback of a single unit, ch2-09, was provided as a 523 Hz tone that sounded every time the unit fired. During session 3, the volume of the audible feedback increased as the unit firing *rate* increased. The gradual smoothing of firing rates in unit ch2-17 over the three sessions parallels the increasing number of lower beta band peaks across the trials shown in Figure 4. Thus the increasing presence of lower beta band peaks would appear to be an indicator of improving performance.

This study is part of a larger series of experiments to provide an adjunctive method that improves the decoding of neural activity recorded from the speech cortex. Regardless of the method of speech decoding, the minimum time segment in which the lower beta band peak can be detected is very important and would have to be as short as possible if beta-band peak detection were to become a method to detect (presumptive) speech onset. In the study with the mute subject, these time segments were mostly between 100 and 200 milliseconds (with one exception). Generally, the stronger the PSD% of the beta band peak at one second, the shorter the minimum time segment (Tables 1 and 2). The latency from firing of the units and resynthesis of the phoneme in the reported studies [4] lies between 30 and 70 ms giving a total time required for decoding of perhaps less than 300 ms when beta band peaks are incorporated into the decoder. These results are corroborated by data in the speaking subjects where the minimum data segments were between 100 and 250 ms as illustrated in Figure 8. In addition, latency between lower beta band peak detection and speech onset can be in the order of 100 to 150 ms as illustrated by the cluster in the lower left corner of Figure 8 when performance is presumably optimal. The cortical firing has a latency of likely 50 to 100 ms before detection at the microphone, so the lower beta band peak is speculated to occur about 50 ms before speech onset.

These results corroborate those of Leventhal et al. [13] who found similar beta band peaks in subjects attempting cued motor tasks. As described in Section 1, their evidence suggests that these beta peaks indicate a stabilized state prior to a cued motor task. In the task described here, the cue was the preparatory phoneme sound (or tone) followed by a “ding” from the computer. The present evidence points towards a similar conclusion, namely, that after the cue, the subject is ready to produce movement of the articulators that are required for successful vocalization. Evidence that this is indeed the case is illustrated by the control periods and the listen periods (where motor activity was presumably absent) that showed a statistically significant dearth of lower beta band peaks as illustrated in Figure 3. Further evidence is shown in Figure 4 where the frequency of lower beta band peaks per session was directly correlated with increasing task success.

The results here are expected to be useful in further development of decoding paradigms for development of speech prostheses. It is expected that in a mute subject, a window of 200, 300, 400, or 500 milliseconds can be moved over the continuous neural data streams to detect lower beta band peaks, while the computer would separate and classify single unit activity from the same data stream. The occurrence of the lower beta band peak could be used to trigger classification of the single unit firing patterns into phonemes, words, or even phrases. A hidden markov model could then resynthesize the speech and output the sound with a near conversational latency. An additional use for these lower beat band peaks could be a binary switch, using the threshold of the peak as the trigger with rejection if the area under the curve does not reach criterion. All these possibilities will be pursued.

## Figures and Tables

**Table 1:** Results of analysis of lower beta band peaks during Silent Speaking. PSD% and frequencies are listed as the average result of the 7 or 8 trials. “Latency” indicates the time (sec) after the speak onset signal in which the beta peak was evident. Minimum time segment indicates the period in which the beta peak was still visible following narrowing of the data segment.

	Number peaks/trial	Latency (sec) from speak onset signal	PSD%	Frequency (Hz)	Minimum data time segment (ms)
IY	5/8	4.63	0.173	14.8	175
AA	6/7	2.75	0.210	14.2	183
OO	5/8	3.85	0.198	15.9	150
Silence	1/8	1.25	0.180	19.2	250

**Table 2:** Results of analysis of lower beta band peaks during Silent Singing. PSD% and frequencies are listed as the average result of all trials. “Latency” indicates the time (sec) after the speak onset signal in which the beta peak was evident. Minimum time segment indicates the period in which the beta peak was still visible.

	No. Peaks during sing	Peak latency (secs)	PSD%	Peak frequency (Hz)	Minimum data time segment (ms)
Tone C5					
Session 1	6	4.92	0.479	15.5	300
Session 2	7	1.44	0.607	14.7	231
Session 3	9	4.14	0.517	15.3	185
Tone C4					
Session 1	3	3.17	0.673	13.8	183
Session 2	4	4.75	0.515	16.3	163
Session 3	8	3.17	0.412	15.4	272
Silence					
Session 1	2	1.50	0.349	16.0	375
Session 2	1	5.50	0.901	15.1	150
Session 3	3	3.30	0.582	14.6	283

## Permission

Permission for implantation of human subjects has been obtained from the Food and Drug Administration, IRB of Neural Signals Inc., and Gwinnett Medical Center where the implantation was performed.

## References

[1] (2008). *Impulse EMG Communication Device*.

[2] Wolpaw J. R., Birbaumer N., McFarland D. J., Pfurtscheller G., Vaughan T. M. (2002). Brain-computer interfaces for communication and control. *Clinical Neurophysiology*.

[3] Miller K. J., Leuthardt E. C., Schalk G. (2007). Spectral changes in cortical surface potentials during motor movement. *Journal of Neuroscience*.

[4] Guenther F. H., Brumberg J. S., Wright E. J. (2009). A wireless brain-machine interface for real-time speech synthesis. *PLoS ONE*.

[5] Donoghue J. P. (2002). Connecting cortex to machines: recent advances in brain interfaces. *Nature Neuroscience*.

[6] Nicolelis M. A. L., Dimitrov D., Carmena J. M. (2003). Chronic, multisite, multielectrode recordings in macaque monkeys. *Proceedings of the National Academy of Sciences of the United States of America*.

[7] Taylor D. M., Tillery S. I. H., Schwartz A. B. (2002). Direct cortical control of 3D neuroprosthetic devices. *Science*.

[8] Hallett M. (1994). Movement-related cortical potentials. *Electromyography and Clinical Neurophysiology*.

[9] Milliken G. W., Stokic D. S., Tarkka I. M. (1999). Sources of movement-related cortical potentials derived from foot, finger, and mouth movements. *Journal of Clinical Neurophysiology*.

[10] Niazi I. K., Jiang N., Tiberghien O., Nielsen J. F., Dremstrup K., Farina D. (2011). Detection of movement intention from single-trial movement-related cortical potentials. *Journal of Neural Engineering*.

[11] Schober T., Wenzel K., Feichtinger M. (2004). Restless legs syndrome: changes of induced electroencephalographic beta oscillations—an ERD/ERS study. *Sleep*.

[12] Grin-Yatsenko V. A., Baas I., Ponomarev V. A., Kropotov J. D. (2009). EEG power spectra at early stages of depressive disorders. *Journal of Clinical Neurophysiology*.

[13] Leventhal D. K., gage G. J., Schmidt R., Pettibone J. R., Case A. C., Berke J. D. (2012). Basal ganglia beta oscillations accompany cue utilization. *Neuron*.

[14] Bartels J., Andreasen D., Ehirim P. (2008). Neurotrophic electrode: method of assembly and implantation into human motor speech cortex. *Journal of Neuroscience Methods*.

[15] Kennedy P. R., Brazino J. (2011). Comparing electrodes for use as cortical control signals: tiny tines, tiny wires or tiny cones on wires: which is best?. *The Biomedical Engineering Handbook*.

[16] Kennedy P. R., Andreasen D. S., Bartels J. (2011). Making a lifetime connection between brain and machine for restoring and enhancing function. *Progress in Brain Research*.

[17] Müller-Putz G. R., Scherer R., Pfurtscheller G., Neuper C. (2010). Temporal coding of brain patterns for direct limb control in humans. *Frontiers in Neuroscience*.

[18] Kennedy P. R., Wichmann T., Wright J. Using cross correlation analysis of recorded units to detect phonemes in human speech cortex.

